# Women's Contexts and Circumstances of Posttraumatic Growth After Sexual Victimization: A Systematic Review

**DOI:** 10.3389/fpsyg.2021.699288

**Published:** 2021-08-26

**Authors:** Marika Guggisberg, Simone Bottino, Christopher M. Doran

**Affiliations:** ^1^Queensland Centre for Domestic and Family Violence Research, School of Nursing Midwifery and Social Sciences, CQUniversity Australia, Perth, WA, Australia; ^2^Cluster for Resilience and Wellbeing, Appleton Institute, CQUniversity Australia, Brisbane, QLD, Australia; ^3^School of Nursing Midwifery and Social Sciences, CQUniversity Australia, Brisbane, QLD, Australia

**Keywords:** posttraumatic growth, rape, recovery, sexual assault, trauma, victimization

## Abstract

Sexual violence is a concerning public health and criminal justice problem. Even though extensive literature has linked sexual victimization to a multitude of mental and physical problems, some victim/survivors recover and are able to lead lives without notable negative impacts. Little is known about women who experienced posttraumatic growth following sexual victimization. This review brings together knowledge accumulated in the academic literature in the past decade. It was informed by the PRISMA-P guidelines. Databases were searched using a combination of keywords to locate original peer-reviewed research articles published between January 2010 and October 2020 focusing on posttraumatic growth following sexual victimization. The initial search identified 6,187 articles with 265 articles being read in full, identifying 41 articles that were included in the analysis. The results suggest that recovery from sexual victimization is possible with the healing process being idiosyncratic. Victim/survivors employed various strategies resulting in higher degrees of functioning, which were termed growth. Following a synthesis of themes that emerged from the thematic analysis, a higher order abstraction, using creative insight through reflexivity, discussions among the research team and consistent interpretation and re-interpretation of the identified themes as a second stage analysis, resulted in the identification of two superordinate topics “relationship to self” and “relationship to others.” Findings indicated that women engaged in deliberate introspection to connect with themselves and utilized altruistic actions and activism in an attempt to prevent further sexual victimization Helping victim/survivors deal with the sexual violence facilitated growth as a collective. We concluded that helping others may be a therapeutic vehicle for PTG. Given research in this area remains in its infancy, further investigation is urgently needed.

## Introduction

Sexual violence, defined as unwanted sexual acts against someone (Ulloa et al., 2016), is a global public health and criminal justice problem (Du Mont et al., 2019). Prevalence rates vary considerably across studies with rates ranging from 10.7 to 21.2% for contact-based sexual violence and up to 15.1% for penetrative sexual offenses against children with consistent findings across international studies that girls are at a considerable higher risk of victimization than boys (Tanaka et al., 2017). Adult victimization of unwanted sexual experiences range from 54% among university students (Campbell et al., 2021) to 19% of victimization by rape for women and 2% for men (Breiding et al., 2014). Studies of sexual violence perpetration indicated that between 25 and 30% of male university students admitted to sexually assaulting a female since the age of 14 years with 68% of men indicating repeat sexual offending (Zinzow and Thompson, 2015). Prevalence rates of sexual violence in current intimate relationships ranged from 18 to 66% (Fernet et al., 2019).

It is important to note that because of multiple victimization definitions, participant characteristics (e.g., age cut offs), recruitment sources and settings, measures of sexual violence (e.g., self-report assessment vs. behavioral descriptors of rape, sexual coercion, non-contact sexual violence) used, prevalence rates tend to vary substantially across studies nationally and internationally, which makes comparisons difficult (Vitek and Yeater, 2020). Regardless of these limitations, studies consistently document high prevalence rates of sexual victimization, defined here as being subjected to inappropriate and/or non-consensual sexual acts, particularly against females (Campbell et al., 2021). Keeping these challenges of research in the area of sexual violence in mind, the following section examines the current knowledge to the major field of child sexual abuse (CSA), adult sexual assault (ASA), revictimization, and impact of sexual victimization.

## Sexual Victimization

It is estimated that 1 in 7 girls and 1 in 25 boys will be sexually abused in childhood (Scoglio et al., 2019). Negative outcomes of child sexual abuse have been extensively documented in the literature (Domhardt et al., 2015). These include mental health problems such as symptoms of anxiety, depression, posttraumatic stress disorder, dissociation as well as health risk behaviors including substance use problems as a coping mechanism (Guggisberg, 2012; Walker et al., 2019). Furthermore, CSA victimization increases the risk of repeat sexual victimization later in life (Messman-Moore and Long, 2003; Hawn et al., 2018).

Prevalence rates differ in studies investigating adult sexual victimization but estimates of nearly 30% have been reported (DeCou et al., 2017). Sexual victimization experiences either in childhood and/or in adulthood has been found to negatively impact the victim/survivor's functioning as well as interpersonal relationships (Vitek and Yeater, 2020).

Lately, researchers have become increasingly interested in understanding sexual revictimization. One consistent finding in the literature is that victim/survivors of sexual abuse tend to be drawn to abusive intimate partners (Vitek and Yeater, 2020). Intimate partner sexual violence (IPSV) has been found to have the most severe and longterm negative effects when compared to other forms such as emotional abuse and/or physical violence (Guggisberg, 2018).

## Impact

Sexual violence has been described as a significant traumatic experience due to the perceived and loss of control over the victimized person's body. This can significantly impact the person's worldview and increase a sense of vulnerability (Ullman and Peter-Hagene, 2014). Strong evidence indicates an association between mental health problems including low self-esteem, anxiety and depression, Posttraumatic Stress Disorder (Scott et al., 2018) and maladaptive coping such as alcohol and/or other drug use (Scoglio et al., 2019; Culatta et al., 2020) and physical health impacts (Guggisberg, 2018). Compared to other forms of interpersonal violence, sexual violence has unique negative impacts including the risk of developing sexually transmitted infections, unwanted pregnancies and other reproductive consequences (Guggisberg, 2021). It is important to note that sexual violence victimization is an idiosyncratic experience and generalization of impacts should be conducted with caution (McFerran et al., 2020), particularly against the background of recent research indicating that posttraumatic growth, defined by Ulloa and colleagues (2016) as “personal transformation that improves quality of life” (p. 293), is possible.

### Posttraumatic Growth

Recent literature indicates that some victim/survivors of sexual violence are able to recover (Domhardt et al., 2015). Research found that psychological and emotional growth is related to positive behavior change (Tedeschi et al., 2018). Several concepts have been used in the literature to describe positive adaptation to CSA victimization including “resilience” (Domhardt et al., 2015) and “posttraumatic growth” (PTG) (Tedeschi et al., 2018). PTG has been conceptualized as a person's positive responses to traumatic events (Muldoon et al., 2020). Typically associated with a person's reconstruction of self and reorientation of priorities and/or values (Tedeschi et al., 2018), with experiences of social support (Muldoon et al., 2020).

Consequently, PTG emphasizes longterm changes following a traumatic event. Tedeschi et al. and associates 2018 distinguished between resilience (i.e., a person's intrapersonal attributes) and PTG, which is the result of permanent change in the aftermath of one or several traumatic events. The vast majority of research to date has focused on medical illness such as cancer (Ochoa Arnedo et al., 2019), natural disasters (e.g., hurricanes) (Hafstad et al., 2011), terror (Eisenberg and Silver, 2011) and war-related violence among affected individuals such as military personnel (Nordstrand et al., 2020). More recently, PTG research included domestic and family violence (DFV) including sexual violence (D'Amore et al., 2018). However, often no distinction was made between the types of violence experiences. Studies fail to distinguish between different forms of intimate partner violence and whether the women had a history of prior victimization.

One review of the literature on sexual victimization and PTG by Ulloa et al. (2016) was identified. Even though the review omitted to use a systematic method and used a gender-neutral approach, findings indicated that victim/survivors reported positive change characterized by engaging in advocacy and activism as a concern for others and factors such as disclosure, social support and spirituality being influencing factors. However, Ulloa et al. (2016) acknowledged the paucity of knowledge on PTG specifically in relation to sexual victimization, which is conceptually different from other forms of trauma and called for further research to inform clinical practice. This systematic review of the literature addresses the gap in the literature on PTG among women who experienced sexual violence.

## The Current Study

### Theoretical Underpinnings

Systematic reviews, like other studies, are underpinned by the researchers' perspectives about the topic under investigation (Alexander, 2020). The authors were cognisant of, and sensitive to, the theoretical context, which is outlined below, that inevitably affected the review process at every stage. Sexual violence is inherently gendered (Guggisberg, 2018). In this regard (Brison, 2020) argued that sexual violence is to be understood as “group-based gender-motivated violence against women” (p. 308). Feminist perspectives draw attention to the gendered nature of violence against women and children particularly in relation to harm sustained from sexual victimization (Alcoff, 2018).

### Rationale

The rationale for this study was that research in the area of sexual victimization predominantly focusses on negative impacts and the long-lasting negative debilitating effects for victim/survivors. While it is justified to continuously highlight the often devastating effects of sexual victimization, it is important to draw attention to the issue of PTG to balance the research and emphasize that some women recover from the trauma and are able to lead satisfactory lives. Given the recent focus on wellbeing and resiliency against the background of adversity, investigating contexts and circumstances of sexual victimization and common factors and behaviors that may be associated with PTG appears particularly relevant. This review's aim was to systematically review the current knowledge on PTG in women who experienced sexual violence in the past. The specific research question guiding this review was: “What are common signs of posttraumatic growth for adult women who experienced sexual violence?” As outlined above this question warrants a review of current knowledge to fill the gap in the literature.

### Methods

The review followed the Preferred Reporting Items for Systematic Reviews and Meta-Analysis Protocols (PRISMA-P) framework (Moher et al., 2015) to identify and analyze published articles specific to sexual victimization and PTG. This review identified qualitative, quantitative and mixed-method studies on sexual violence and PTG.

#### Procedure

A systematic search of research studies was undertaken after scoping searches focused on PTG following sexual victimization. The research team, consisting of two experienced senior researchers who previously conducted systematic reviews and a student researcher developed a research protocol. All decisions including aim, criteria for inclusion, search strategies, key words and concepts, methods of review, quality assessment and data analysis, were made in collaboration, which included reviews and revisions until agreement was reached.

#### Literature Search Strategy

The second author, under close supervision, searched the following databases: Ebscohost, Google Scholar, PsycINFO, Medline, OVID, and Web of Science to identify relevant records. Only peer-reviewed articles published in English were included using multiple keywords sets and synonyms (singular and plural forms and different spelling) such as child sexual abuse, adult survivors, healing, recovery, posttraumatic growth, sexual assault, sexual violence, and rape. Various search operators including truncation and wildcard symbols were used to identify studies until no new articles were found.

#### Inclusion Criteria

The following inclusion criteria were used to identify relevant articles on PTG following sexual victimization: (a) and peer reviewed academic articles of original research in English, (b) published between January 2010 and October 2020, (c) female victim/survivors of sexual violence who reported PTG. To be included, studies had to be conducted on sexual victimization and PTG with female participants.

#### Exclusion Criteria

When studies included other forms of DFV (e.g., emotional abuse, physical violence), only information about sexual violence was considered. Studies were excluded on child abuse and/or intimate partner violence if no sexual violence measure was reported. Studies with samples of only male victims of sexual violence and participants where results were not distinguishable by gender were also excluded from this review. Furthermore, commentaries, government reports, newspaper and web-based articles, brochures, newsletters, conference papers and student theses were excluded along with research articles published in a language other than English.

Nearly 9,200 records were identified with 2,987 duplicates leading to 6,187 records. As suggested by Belur et al. (2021), we attempted to reduce errors due to fatigue effects by undertaking the initial identification process in “small batches.” Titles and abstracts were screened for eligibility following the removal of duplicates. Furthermore, a cautious approach was taken to reduce the potential of excluding a possible relevant article. If the decision for exclusion at this stage was unclear, the record was included. The interpretation of this bivariant screening process (i.e., decision whether to include or exclude a study) was closely observed by the first and second author using a constant comparison approach. The number of studies identified as being included varied slightly when results were compared. Consistent reflections and discussions about how to understand the task of applying the criteria enhanced the confidence in this process and reduced the risk of bias.

Two hundred and sixty-five full text articles were read and discussed by the first and second author to confirm inclusion in the review. Results were compared, reasoning for decisions included individual reflection on decisions and disagreements reconciled. Clarity on inclusion and exclusion criteria was enhanced and carefully recorded using an audit trail. Full text reviews resulted in the exclusion of 218 articles for various reasons including unclear information on sexual violence and/or female victim/survivors. Decisions were made in collaboration and discrepancies during this process were resolved through discussions using individual reflection and interpretation of the specific inclusion criteria. At this stage, the researchers applied their clinical knowledge of the research topic and well developed reflective practice on the final decisions for inclusion of articles, which further enhanced reliability. This was important as accuracy and consistency of inclusion decisions are influenced by the researchers' knowledge and understanding of the subject matter (Belur et al., 2021). Before articles were included in the final review, an independent quality appraisal by the first and second author was conducted utilizing the standardized quality assessment Mixed Methods Appraisal Tool (MMAT) for systematic reviews (Hong et al., 2018), which has showed content validity and reliability (Hong et al., 2019).

**Figure 1:**
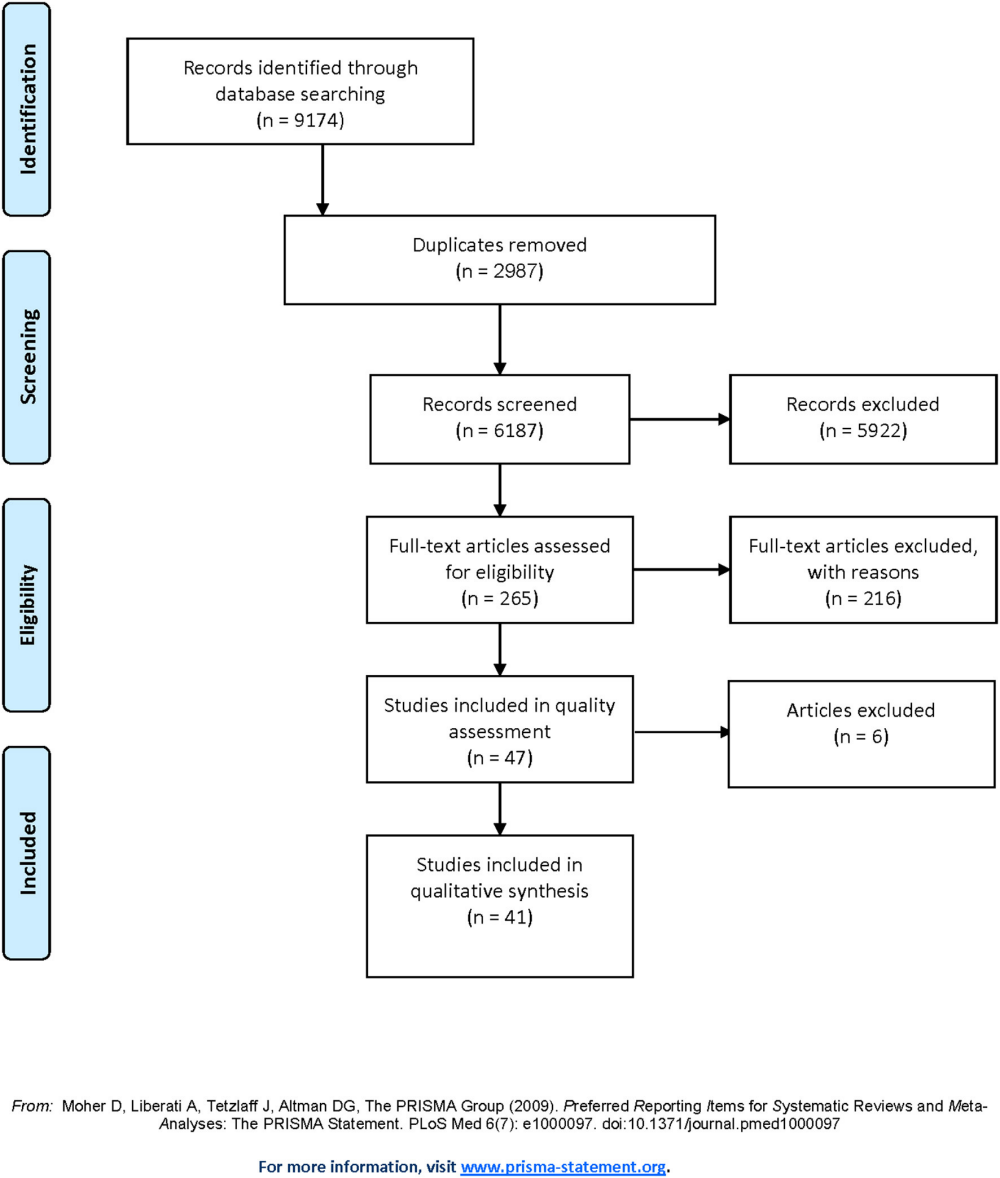
PRISMA flow diagram of screening process.

Each study received a specific quality rating score, which ranged from 0 to 6. Studies with total scores between 4 and 6 were considered high quality with low risk of bias. Those with a score of 3 were considered borderline and discussed by the researchers to arrive at an agreement of inclusion or exclusion. Studies given a score of 2 or less were automatically excluded for lack of methodological confidence (e.g., no identified research question, low quality of methods and/or measurement, and inappropriate source population). Given the potential risk of bias, it was mutually agreed to exclude 6 studies from the review. Meticulous record keeping ensured inter-rater agreement during the data extraction and assessment process, as illustrated in Figure 1.

#### Data Analysis

Data were contextualized and analyzed using a thematic analysis approach (Braun and Clarke, 2006). After the development of initial themes, we discussed the themes on an iterative basis before interpreting our finding in an attempt to understand PTG conceptualizations at a more abstract level that enables generalization of our findings (Eakin and Gladstone, 2020). Generating generalizable concepts, according to Eakin and Gladstone (2020) is a key objective of quality analysis. This creative insight is a result of reflexivity, discussion among the researchers and consistent interpretation and re-interpretation of the identified themes.

## Findings

### General Study Characteristics

A summary of general study characteristics is presented in Table 1. All 41 articles reported on PTG following female sexual victimization. The sample sizes of studies ranged from 2 to 39,703 with 19 (48.8%) studies having used qualitative, 19 (46.3%) quantitative, and 3 (7.3%) mixed-methods. The vast majority (63.4%) of studies were conducted in the USA (Cole and Lynn, 2010; Singh et al., 2010; Drauker et al., 2011; Warner Stidham et al., 2012; Williams and Nelson-Gardell, 2012; Wilson et al., 2012; Arias and Johnson, 2013; Davidson et al., 2013; McClain and Frederick Amar, 2013; Foster and Hagedorn, 2014; Ullman, 2014; Bryant-Davis et al., 2015; Kelley and Gidycz, 2015; Simon et al., 2015; Crews et al., 2016; Hartley et al., 2016; Hitter et al., 2017; Kerlin and Sosin, 2017; Smigelsky et al., 2017; Barnett and Maciel, 2019; Catabay et al., 2019; Kirkner and Ullman, 2019; Lahav et al., 2019; Nelson et al., 2019; Saint Arnault and Sinko, 2019; Strauss Swanson and Szymanski, 2020) followed by European studies with 9.8% (Phanichrat and Townshend, 2010; Whitelock et al., 2013; Perez-Gonzalez et al., 2017; Anderson et al., 2019), and two in South Africa (Phasha, 2010; Walker-Williams et al., 2012). One study each was from Australia (Vilencia et al., 2013), Bangladesh (Kaiser and Sinanan, 2020), Brazil (Pessoa et al., 2017), India (George and Bance, 2019), Israel (Kaye-Tzadok and Davidson-Ard, 2016), Korea (Ha et al., 2019), Nepal (Volgin et al., 2019), Rwanda (Zraly et al., 2013), and Taiwan (Wang and Heppner, 2011). Twenty-two studies (53.7%) placed specific focus on CSA while six studies (14.6%) examined lifetime sexual victimization and some used specific participants such as sexual trafficking and wartime victim/survivors. The vast majority of studies were cross-sectional (85.4%). Six studies (14.6%) used a longitudinal design (see Table 1).

**Table 1:** Study characteristics.

**Authors (Year)**	**Location/Country**	**Study design**	**Sample**	**Comments**
Anderson et al. (2019)	Europe	Mixed-methods	104 women	War rape
Arias and Johnson (2013)	USA	Qualitative	10 women	History of CSA
Barnett and Maciel (2019)	USA	Quantitative	199 women	Lifetime victimization
Bryant-Davis et al. (2015)	USA	Quantitative	252 women	African-American participants
Catabay et al. (2019)	USA	Quantitative	94 women	Adult victimization
Cole and Lynn (2010)	USA	Quantitative	105 women	Unwanted sex experiences
Crews et al. (2016)	USA	Qualitative	8 females	Lifetime victimization
Davidson et al. (2013)	USA	Quantitative	503 women	lifetime victimization
Drauker et al. (2011)	USA	Quantitative	95 participants	History of CSA; 48 women 47 men
Foster and Hagedorn (2014)	USA	Qualitative	21 participants	Adolescents, 18 female 3 male
George and Bance (2019)	India	Quantitative	132 females	History of CSA, 15–24 years
Ha et al. (2019)	Korea	Quantitative	32 participants	Child and adult victimization
				29 women, 3 men
Hartley et al. (2016)	USA	Qualitative	6 women	History of CSA (family member)
Hitter et al. (2017)	USA	Qualitative	8 women	History of CSA
Kaiser and Sinanan (2020)	Bangladesh	Qualitative	12 girls	History of CSA (13 years old)
Kaye-Tzadok and Davidson-Ard (2016)	Israel	Quantitative	100 women	History of CSA
Kelley and Gidycz (2015)	USA	Quantitative	135 women	Adolescent/adult victimization
Kerlin and Sosin (2017)	USA	Qualitative	10 women	history of CSA
Kirkner and Ullman (2019)	USA	Quantitative	983 women	Lifetime victimization
Lahav et al. (2019)	USA	Quantitative	95 women	History of CSA (revictimisztion)
McClain and Frederick Amar (2013)	USA	Qualitative	12 women	History of CSA
Nelson et al. (2019)	USA	Quantitative	292 participants	History of CSA
				247 women, 23 men (3 “other,” 19 missing)
Perez-Gonzalez et al. (2017)	Spain	Quantitative	97 adolescents	History of CSA (revictimization)
				3 girls, 24 boys (12–17 years)
Pessoa et al. (2017)	Brazil	Qualitative	7 participants	History of CSA
				6 girls, 1 boy (12–15 years old)
Phanichrat and Townshend (2010)	UK	Qualitative	7 participants	4 women, 3 men
Phasha (2010)	South Africa	Qualitative	4 women	History of CSA (revictimization)
Saint Arnault and Sinko (2019)	USA	Mixed-methods	206 women	History of CSA
Simon et al. (2015)	USA	Mixed-methods	118 participants	History of CSA (revictimization)
				87 girls, 31 boys (8–11 years)
Singh et al. (2010)	USA	Qualitative	13 women	History of CSA
Smigelsky et al. (2017)	USA	Qualitative	9 women	Refugees
Strauss Swanson and Szymanski (2020)	USA	Qualitative	16 participants	13 females 3 genderqueer
Ullman (2014)	USA	Quantitative	1,863 women	Lifetime victimization
Vilencia et al. (2013)	Australia	Qualitative	2 women	Rape (revictimisation)
Volgin et al. (2019)	Nepal	Qualitative	26 adolescent girls	Sex trafficking, revictimization
Walker-Williams et al. (2012)	South Africa	Quantitative	60 women	History of CSA
Wang and Heppner (2011)	Taiwan	Qualitative	10 women	History of CSA
Warner Stidham et al. (2012)	USA	Qualitative	121 participants	Lifetime, 64 women 57 men
Whitelock et al. (2013)	UK	Quantitative	47,869 participants	History of CSA
				39,793 women 8,076 men
Williams and Nelson-Gardell (2012)	USA	Quantitative	237 participants	History of CSA
				190 women 47 men
Wilson et al. (2012)	USA	Quantitative	32 women	Lifetime victimization
Zraly et al. (2013)	Rwanda	Qualitative	4 women	CSA rape (revictimization)

**Figure 2:**
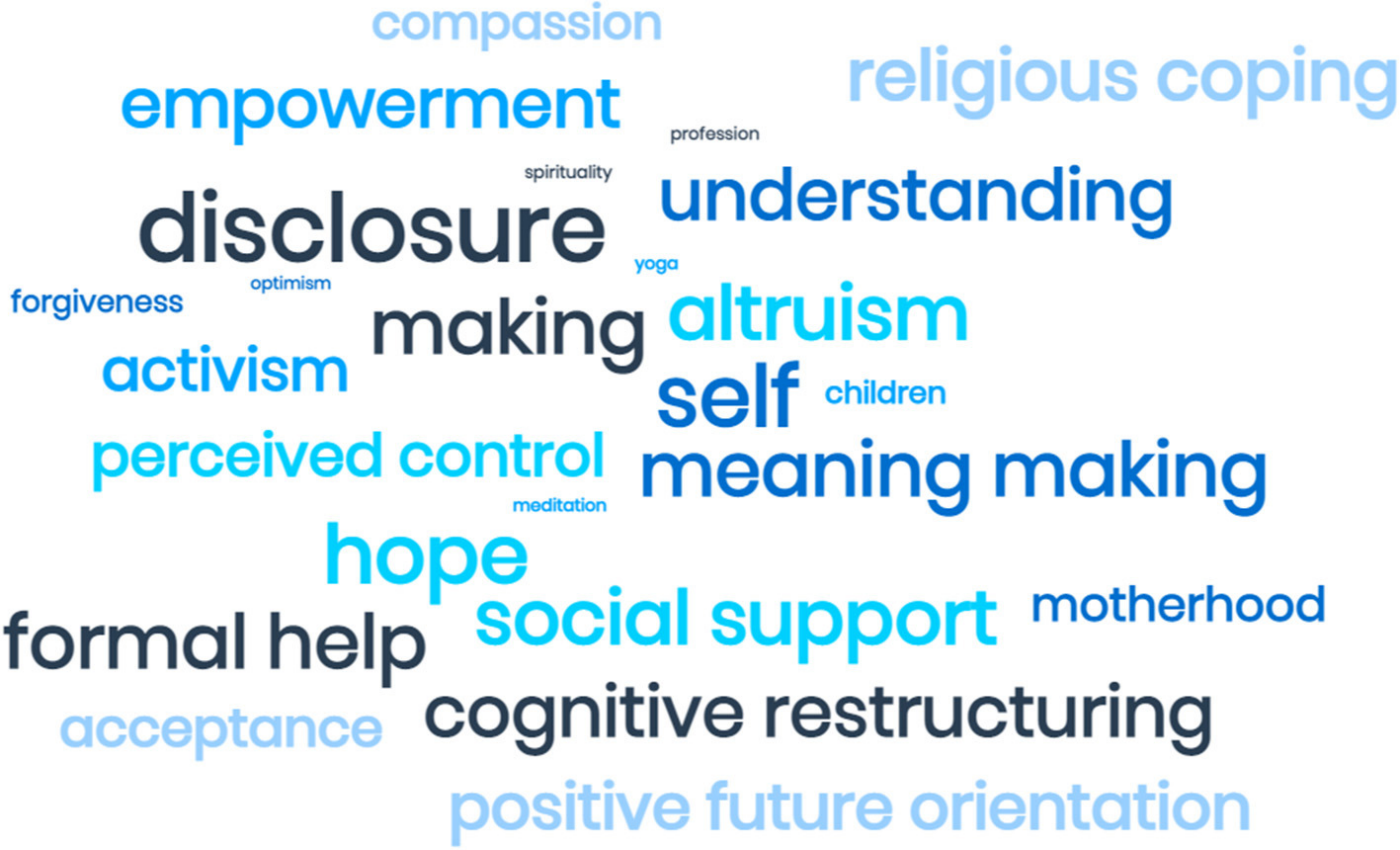
Visualization of findings.

The thematic analysis found that all studies reported some form of PTG occurring with variations according to study focus and revictimization experiences. Within the studies, noteworthy inconsistencies in conceptualizations of PTG as well as varying methods utilized to describe and assess PTG were observed. Researchers conceptualized PTG by obtaining an overall rating using specific measures such as the PTG Inventory (Tedeschi and Calhoun, 1996), its short form (Cann et al., 2010), whereas others assessed PTG through women's subjectively held attitudes, actions, and evaluations of their recovery. Participants agreed that their sexual victimization experiences should not be forgotten. The studies indicated a willingness to not let the past control their present or future and the decision to actively engage in different ways in an attempt to get beyond the traumatic experiences, often coupled with faith, positive rather than negative thinking, and deliberate actions such as help seeking, activism and advocacy (**Figure 2**).

Figure 2 illustrates the words/phrases used by the studies to describe PTG. Numerous words pointed to introspection and efforts to reaching out. Further interpretation and reflective discussions resulted in two abstract overarching themes “relationship to self” and “relationship to others” (see Table 2), which will be discussed below.

**Table 2:** Superordinate and sub themes.

**Authors (Year)**	**Superordinate**	**Subthemes**
Anderson et al. (2019)	Self	Cognitive restructuring (positive reinterpretation), positive future orientation
Arias and Johnson (2013)	Self	Acceptance, compassion, forgiveness, future orientation, cognition, control
	Others	Disclosure (confronting the abuser), formal help-seeking, altruism (helping others)
Barnett and Maciel (2019)	Self	Cognitive restructuring, optimism
Bryant-Davis et al. (2015)	Self	Religious coping
	Other	Social support (family, friends, religious service attendance)
Catabay et al. (2019)	Other	Social support (partner, family, friends, current romantic partners)
Cole and Lynn (2010)	Self	Acceptance
	Others	Disclosure
Crews et al. (2016)	Self	Acceptance, self-compassion, self-kindness, yoga, mindfulness, spirituality
Davidson et al. (2013)	Self	Forgiveness, perceived control
Drauker et al. (2011)	Self	Meaning making, religious coping
	Others	Disclosure, informal and formal help-seeking, altruism
Foster and Hagedorn (2014)	Self	Self-perception, hope, positive future orientation
	Others	Formal help-seeking, altruism, enter helping profession
George and Bance (2019)	Self	Cognitive reconstruction
Ha et al. (2019)	Self	Forgiveness
Hartley et al. (2016)	Self	Religious coping, meaning making, self-perception, empowerment, future orientation
	Others	Disclosure, interpersonal relationships (children)
Hitter et al. (2017)	Self	Self-understanding (identity)
	Others	Disclosure, formal help seeking, altruism (create prevention group)
Kaiser and Sinanan (2020)	Self	Hope, future orientation, motherhood (healing)
	Others	Protect children (educate about sexual violence)
Kaye-Tzadok and Davidson-Ard (2016)	Self	Hope, cognitive restructuring, meaning making
Kelley and Gidycz (2015)	Self	Self-perception, meaning making
Kerlin and Sosin (2017)	Self	Self-image, religious coping
	Others	Formal help-seeking (recovery group), altruism (helping others)
Kirkner and Ullman (2019)	Self	Perceived control, religious coping
Lahav et al. (2019)	Self	Hope, optimism, positive future orientation
	Others	Formal help seeking
McClain and Frederick Amar (2013)	Self	Hope, positive future orientation,
	Others	Disclosure (breaking the silence), motherhood, altruism (helping others)
Nelson et al. (2019)	Self	Self-understanding
	Others	Disclosure
Perez-Gonzalez et al. (2017)	Self	Self-compassion, positive future orientation
	Others	Formal help-seeking (therapy)
Pessoa et al. (2017)	Self	Self-concept, empowerment, positive future orientation
	Others	Altruism (providing social support)
Phanichrat and Townshend (2010)	Self	Acceptance, hope, cognitive restructuring, meaning making, religious coping
	Others	Join support group, disclosure (internet forum)
Phasha (2010)	Self	Hope, empowerment, positive future orientation, religious coping, meaning making
	Others	Disclosure, informal and formal help-seeking
Saint Arnault and Sinko (2019)	Self	Acceptance, hope, cognitive restructuring, empowerment, future orientation
Simon et al. (2015)	Self	Self-concept, cognitive restructuring, empowerment
	Others	Disclosure, informal social support (partner, family)
Singh et al. (2010)	Self	Meaning-making, hope, perceived control, self-confidence, empowerment
	Others	Disclosure (breaking the silence), social support (family, friends), yoga, meditation
Smigelsky et al. (2017)	Self	Perceived control, meaning making, religious coping
	Others	Motherhood
Strauss Swanson and Szymanski (2020)	Self	Meaning-making, self-concept, empowerment, perceived control
	Others	Disclosure, altruism (helping others), activism (speaking out), helping profession
Ullman (2014)	Self	Perceived control, adaptive individual coping, attitudes and perception
	Others	Disclosure, formal help-seeking
Vilencia et al. (2013)	Self	Meaning-making, self-compassion, self-perception, hope, cognitive restructuring
	Others	Disclosure, formal help-seeking
Volgin et al. (2019)	Self	Cognitive restructuring, self-compassion, hope, empowerment
	Others	Social support, altruism (helping others)
Walker-Williams et al. (2012)	Self	Self-views, spirituality
	Others	Disclosure, informal and formal help-seeking
Wang and Heppner (2011)	Self	Self-acceptance, religious coping, positive future orientation, self-education
	Others	Disclosure, help-seeking (family), altruism (helping others)
Warner Stidham et al. (2012)	Self	Meaning-making, cognitive restructuring, empowerment, self-education
	Others	Motherhood (protecting children), disclosure, informal and formal help-seeking, advocacy (speaking out) altruism (helping others), activism (public engagement)
Whitelock et al. (2013)	Self	Self-views, reorientation (values, priorities),
	Others	Informal social support, formal help-seeking
Williams and Nelson-Gardell (2012)	Self	Expectancies, hope, education
	Others	Disclosure, social support (family), activism
Wilson et al. (2012)	Self	Emotion regulation, cognitive restructuring, self-awareness, meditation
	Others	Disclosure, social support, therapy
Zraly et al. (2013)	Self	Self-understanding, reorientation (values – hope, gratitude), religious coping
	Others	Mothering (relationship with children, living for children, protecting children

### Relationship to Self

The majority of studies revealed that women learned to connect with themselves in new ways upon engaging in deliberate introspection. Many studies reported how participants utilized trauma-related affective and cognitive coping mechanisms including searching for meaning, taking control, and engaging in decision making processes. Numerous studies reported that after becoming aware of their emotions and thoughts women changed effortful cognitive and behavioral avoidance behaviors and engaged in mobilizing effortful strategies that resulted in a reduction of negative symptoms and increased their functioning ability and quality of life. Participants focusing on themselves, engaging in cognitive and behavioral techniques have been found to experience empowerment and recovery from sexual victimization. These insights developed in the context of informal social support and therapy after professional help seeking efforts.

### Relationship to Others

Most studies indicated that women sought relationships with others. Engaging with others after sexual victimization can be particularly challenging, which is why some women prefer a coping strategy that does not involve relationship with others (Ha et al., 2019). The reason for some women's difficulties establishing relationships with others after sexual victimization is the impact of secrecy and betrayal, particularly if the perpetrator was a biological father (Guggisberg, 2017). It is important to note that the decision not to seek social support should be seen as an expression of perceived control rather than a sign of helplessness (Wasco, 2003).

Findings of this review indicated that the vast majority of women disclosed their sexual victimization and sought informal and/or formal support. Women in several studies indicated that they experienced a sense of belonging and recovery through social engagement such as music, yoga and religious service attendance. The studies reported that the experience of interaction was beneficial in that it was perceived as empowering and enhanced self-understanding. Social engagement in the aftermath of sexual victimization has been found to assist women in establishing safety and stability followed by reconstructing the trauma narrative through means of music and other forms of creative therapy (Herman, 2015). Herman (2015) advocated for meaning-making and using reflection as an integrated effort to re-establish ownership of self. The process of reconnecting with self, integrating the victimization experiences, is an integral part of the recovery process, which allows victim/survivors to experience self-compassion and grief whereby connecting with the traumatic experiences emotionally to self-sooth and physically reconnect with their body. Religious engagement, music and yoga have been mentioned by several studies, that align with Herman's (2015) theoretical explanation of integrating the women's private and public self.

Furthermore, women actively engaged in altruism by helping other victim/survivors Importantly, it has been recognized that victim/survivors themselves are excellently situated to engage in peer support as they understand the importance of choice, which, unsurprisingly, has been associated with experiences of recovery from trauma (Sweeney et al., 2018).

### Combining the Relationships

Most studies indicated that engaging with oneself and others was a mutually inclusive process. Experiencing inner healing led to social engagement, which furthered feelings of empowerment and control in a bidirectional fashion. Sweeney et al. and colleagues 2018 explained this observation with what they referred to as the “triangle of wellbeing,” an “interaction between the social, personal and biological realms” (p. 319). Indeed, sexual victimization has been found to have an impact on neurobiological processes particularly if retraumatization occurs (Guggisberg, 2017; Sweeney et al., 2018). In this regard, Guggisberg (2017) emphasized the importance of therapy particularly in relation to intergenerational healing and recovery.

Taken together, women who experience PTG are generally utilizing a combination of strategies that can be subsumed under relationship to “self” and “others.” Victim/survivors have in common the desire to regain control over their bodies, minds, and regaining a sense of trust in people. This inner struggle and pursuit of integrating their sexual victimization experience into a new sense of self and with others can be explained as PTG with a deliberate choice to take proactive steps rather than escape and avoidance strategies.

Once the realization of choice and decision making is achieved, the individual is free to open their mind and facing the option of choosing to change the status quo (Schick, 1998). Schick (1998) indicated: “we have options… to make a choice… to choose is to come to want to take this or that option we have. To choose is to come to want something that is an option for us… we must try to think of wanting in anon-hedonic way, in a way that allows for our wanting what we know will be painful” (p. 11). For a victim/survivor of sexual violence this means confronting coping behaviors associated with the sexual trauma, which Schick (1998) conceptualized as an “inner changing” (p. 12) whereby things appear in a new light and understanding reasons behind the actions that are possibilities to be chosen. Similarly, Bermudez (2018) indicated that motivation for a particular change in behavior over another can be explained as exercising self-control. Being aware of various options and the possibility to choose, is engaging in conscious decision making, which entails thinking about a desired outcome and working toward an identified goal (Bermudez, 2018). Several studies indicated the importance of self-control, empowerment and positive future orientation (see Table 2).

### Speaking Out

Relationships with self and others were discussed in 30 studies where participants indicated how they were able to engage with others after self-discovery. Interpersonal relationships were described as helpful in the recovery process for the women and many indicated the need for breaking the silence about their sexual victimization as indicated in 18 studies, even specifically confronting their abuser (Arias and Johnson, 2013), or helping others by various means such as creating a specific recovery group (Kerlin and Sosin, 2017) but also prevention group (Hitter et al., 2017) and entering a helping profession (Foster and Hagedorn, 2014). Victim/survivors seem to enact agency by speaking out about their experiences of sexual harm along with the desire to speak for others who share the same experiences. Talking about sexual victimization experiences publicly has become a movement, which creates collectivity (Alcoff, 2018).

Given the risk of non-supportive responses including victim-blaming statements following disclosure of sexual victimization (Bhuptani et al., 2019), many women feel safer to voice their experiences anonymously online (Mendes et al., 2019). Women disclosing their sexual victimization using social media have reported experiencing liberation and feeling empowered in having their voices heard Positive reactions to disclosures of sexual victimization have been related to perceived control and increased feelings of self-efficacy as described by several studies. In this regard, it is important to note that even negative responses to disclosure have resulted in increased perceived control and positive coping efforts (Ullman and Peter-Hagene, 2014). Digital disclosure of sexual victimization has been described as being part of a broader movement of speaking out such as #Me Too and #BeenRapedNeverReported, which has been associated with reduced risk of revictimization and improved psychological health (Moors, 2013). A discussion about the possible challenges of using social media as a means of disclosure is beyond the scope of this paper. However, making allegations of sexual violence against a specific person can be ethically and legally problematic (Salter, 2013).

Six studies specifically mentioned women engaging preventatively to ensure their children were protected from sexual violence. Not only did the women recognize their potential and unique position in protecting their children from child sexual abuse (Guggisberg et al., 2021), their children from child sexual abuse but their sense of control and personal agency was related to a positive future orientation and increased confidence, as discussed by nearly half of the studies. To take control over their lives in an attempt to overcome the traumatic events tended to lead to experiencing a sense of agency, resulting in altruism and advocacy behaviors. As discussed above, online communities have become increasingly used by women to discuss their sexual victimization, which have not only been recognized as an avenue for activism but an important support mechanism, especially for women who are geographically isolated as online platforms are easily accessible and contribute to a sense of connectedness (Serisier, 2019).

### Strengths and Limitations

This review substantially advances knowledge on PTG among female victim/survivors of sexual violence. It not only provides an overview and summary of indicators associated with PTG but goes beyond by theorizing about victim/survivors' relationship to self and others. Regardless, the results of this review should be considered in the light of the following limitations: Firstly, focus was placed on female victim/survivors of sexual violence, which means findings may vary for males. Perhaps a separate study involving males would be helpful in assessing this point. Furthermore, even though a large amount of studies was identified published between 2010 and 2020, it is possible that some information was missed due to the publication date limits set. Additionally, as Belur et al. and colleagues 2021 argued, subjectivity must be acknowledged, which may impact the selection of records included in the review. The studies identified in this paper only represent peer-reviewed original research publications; information presented in theses, government reports and conference papers may have been omitted due to the restrictive inclusion criteria. Consequently, generalizability may be limited by the researchers' interpretations of the subject matter along with varying cultural contexts that were not comprehensively explored in this review. Therefore, further research may benefit from removal of time limits, the inclusion of publications other than peer-reviewed studies only, an examination of specific cultural implications, not only for female but male victim/survivors and those identifying as non-cisgender individuals along with quantitative studies that use a meta-analysis approach. Further research should explore PTG experiences among women who were subjected to multiple types of sexual victimization (i.e., in childhood, adolescence, and/or adulthood) in their lifetime to better understand the specific implications of PTG associated with revictimization. There is a need for further research focusing specifically on different types of sexual victimization in childhood vs. adolescence and adulthood. Age-related research may uncover different coping and help-seeking strategies in relation to PTG.

## Conclusion

This review systematically and methodically examined the literature on the two constructs sexual violence victimization and PTG among female victim/survivors. Findings revealed that recovery from sexual victimization is possible as the 41 analyzed articles indicated. The numerous identified themes suggested that the healing process is idiosyncratic and an individual journey for victim/survivors and many different strategies were employed by the women who participated in the studies that were reviewed. Common themes involved women's self-reflection and meaning making activities, resulting in higher degrees of functioning and positive change, which were termed growth. Cognitive appraisal involved having control and decision making abilities that were experienced as empowering. The articles emphasized the interaction between women's search for meaning, valuing themselves and finding a new purpose and meaning in life and analysis revealed the importance of the notions of control and decision making. Following a synthesis of themes that emerged, a higher order abstraction resulted in the identification of two superordinate topics, which we categorized as “relationship to self” and “relationship to others,” leading to altruistic actions and activism in an attempt to prevent further sexual victimization. Helping victim/survivors deal with the sexual violence facilitated growth as a group, which points to the conclusion that helping others may be a therapeutic vehicle for PTG individually as well as collectively. We recommend developing interventions that reinforce the themes inherent in this review. Furthermore, given that research in this area remains in its infancy, further investigation is urgently needed.

## Data Availability Statement

The original contributions presented in the study are included in the article/supplementary material, further inquiries can be directed to the corresponding author/s.

## Author Contributions

MG and SB undertook the analysis and interpreting of the data with discussions of the texts and analytical thoughts involving all authors. MG contributed to the initial draft with the other authors critically reviewing numerous drafts, providing commentary on revisions during the pre-publication stage. All authors were responsible for the conceptualization and writing of this article.

## Conflict of Interest

The authors declare that the research was conducted in the absence of any commercial or financial relationships that could be construed as a potential conflict of interest.

## Publisher's Note

All claims expressed in this article are solely those of the authors and do not necessarily represent those of their affiliated organizations, or those of the publisher, the editors and the reviewers. Any product that may be evaluated in this article, or claim that may be made by its manufacturer, is not guaranteed or endorsed by the publisher.

## References

[B1] Alcoff L. M. (2018). Rape and Resistance: Understanding the Complexities of Sexual Violation. Medford, MA: Polity Press.

[B2] Alexander P. A. (2020). Methodological guidance paper: the art and science of quality systematic reviews. Rev. Educ. Res. 90, 6–23. 10.3102/0034654319854352

[B3] Anderson K. Delic A. Komproe I. Avidibegovic E. van Ee E. Glaesmer H. (2019). Predictors of posttraumatic growth among conflict-related sexual violence survivors from Bosnia and Herzegovina. Confl. Health 13, 1–11. 10.1186/s13031-019-0201-5 31171935 PMC6549258

[B4] Arias B. Johnson C. V. (2013). Treatment of childhood sexual abuse survivors: voices of healing and recovery from childhood sexual abuse. J. Child Sex. Abus. 22, 822–841. 10.1080/10538712.2013.830669 24125084

[B5] Barnett M. D. Maciel I. V. (2019). Counterfactual thinking among victims of sexual assault: Relationships with posttraumatic stress and posttraumatic growth. J. Interpers. Violence. 10.1177/0886260598952629. [Epub ahead of print]. 31142184

[B6] Belur J. Tompson L. Thornton A. Simon M. (2021). Interrater reliability in systematic review methodology: exploring variation in coder decision-making. Sociol. Methods Res. 50, 837–865. 10.1177/0049124118799372

[B7] Bermudez J. L. (2018). Frames, rationality, and self-control, in Self-control, Decision Theory, and Rationality: New Essays, eds Bermudez J. L. (Cambridge, UK: Cambridge University Press), 179–202. 10.1017/9781108329170.009

[B8] Bhuptani P. H. Kaufman J. S. Messman-Moore T. L. Gratz K. L. DiLillo D. (2019). Rape disclosure and depression among community women: the mediating roles of shame and experiential avoidance. Violence Against Women 25, 1226–1242. 10.1177/1077801218811683 30474500

[B9] Braun V. Clarke V. (2006). Using thematic analysis in psychology. Qual. Res. Psychol. 3, 77–101. 10.1191/1478088706qp063oa

[B10] Breiding M. J. Smith S. G. Basile K. C. Walters M. L. Chen J. Merrick M. T. (2014). Prevalence and characteristics of sexual violence, stalking, and intimate partner violence victimization: National Intimate Partner and Sexual Violence Survey United States, 2011. Centers for Disease Control. Available online at: http://origin.glb.cdc.gov/mmwr/ss6308 (accessed September 15, 2020). PMC4692457 25188037

[B11] Brison S. J. (2020). Can we end the feminist 'sex wars' now? Comments on Linda Martin Alcoff, Rape and resistance: understanding the complexities of sexual violation. Philos. Stud. 177, 303–309. 10.1007/s11098-019-01392-z

[B12] Bryant-Davis T. Ullman S. Tsong Y. Anderson G. Counts P. Tillman S.. (2015). Healing pathways: longitudinal effects of religious coping and social support on PTSD symptoms in African American sexual assault survivors. J. Trauma Dissociation 16, 114–128. 10.1080/15299732.2014.969468 25387044 PMC4286490

[B13] Campbell J. C. Sabri B. Budhathoki C. Kaufman M. R. Alhusen J. Decker M. R. (2021). Unwanted sexual acts among university students: correlates of victimization and perpetration. J. Interpers. Violence 36, 504–526. 10.1177/0886260517734221 29294944 PMC5878971

[B14] Cann A. Calhoun L. G. Tedeschi R. G. Taku K. Vishnevsky T. Triplett K. N.. (2010). A short form of the Post Traumatic Growth Inventory. Anxiety Stress Coping 23, 127–137. 10.1080/10615800903094273 19582640

[B15] Catabay C. J. Stockman J. K. Campbell J. C. Tsuyki K. (2019). Perceived stress and mental health: the mediating roles of social support and resilience among black women exposed to sexual violence. J. Affect. Disord. 259, 143–149. 10.1016/j.jad.2019.08.037 31445340 PMC6791774

[B16] Cole A. S. Lynn S. J. (2010). Adjustment of sexual assault survivors: hardiness and acceptance coping in posttraumatic growth. Imagin. Cogn. Pers. 30, 111–127. 10.2190/IC.30.1.g

[B17] Crews D. A. Stolz-Newton M. Grant N. S. (2016). The use of yoga to build self-compassion as a healing method for survivors of sexual violence. J. Religion Spiritual. Soc. Work Soc. Thought 35, 139–156. 10.1080/15426432.2015.1067583

[B18] Culatta E. Clay-Warner J. Boyle K. M. Oshri A. (2020). Sexual revictimization: a routine activity theory explanation. J. Interpers. Violence, 35, 1–25. 10.1177/0886260517704962 29294726

[B19] D'Amore C. Martin S. L. Wood K. Brooks C. (2018). Themes of healing and posttraumatic growth in women survivors' narratives of intimate partner violence. J. Interpers. Violence 36, NP2697–NP2724. 10.1177/0886260518767909 29642769

[B20] Davidson M. M. Lozano M. Cole B. P. Gervais S. J. (2013). Associations between women's experiences of sexual violence and forgiveness. Violence Victims 28, 1041–1053. 10.1891/0886-6708.VV-D-12-00075 24547679

[B21] DeCou C. R. Cole T. T. Lynch S. M. Wong M. M. Matthews K. C. (2017). Assault-related shame mediates the association between negative social reactions to disclosure of sexual assault and psychological distress. Psychol. Trauma Theory Res. Pract. Policy 9, 166–172. 10.1037/tra0000186 27607768

[B22] Domhardt M. Muenzer A. Fegert J. M. Goldbeck L. (2015). Resilience in survivors of child sexual abuse: a systematic review of the literature. Trauma Violence Abuse 16, 476–493. 10.1177/1524838014557288 25389279

[B23] Drauker C. B. Martsolf D. S. Roller C. Knapik G. Ross R. Warner Stidham A. (2011). Healing from childhood sexual abuse: a theoretical model. J. Child Sex. Abus. 20, 435–466. 10.1080/10538712.2011.588188 21812546 PMC3970162

[B24] Du Mont J. Johnson H. Hill C. (2019). Factors associated with posttraumatic stress disorder symptomatology among women who have experienced sexual assault in Canada. J. Interpers. Violence. 10.1177/0886260519860084 31288606

[B25] Eakin J. M. Gladstone B. (2020). “Value-adding” analysis: doing more with qualitative data. Int. J. Qual. Methods 19, 1–13. 10.1177/1609406920949333

[B26] Eisenberg N. Silver R. C. (2011). Growing up in the shadow of terrorism: youth in America after 9/11. Am. Psychol. 66, 468–481. 10.1037/a0024619 21823779

[B27] Fernet M. Lapierre A. Herbert M. Cousineau M.-M. (2019). A systematic review of literature on cyber intimate partner victimization in adolescent girls and women. Comput. Human Behav. 100, 11–25. 10.1016/j.chb.2019.06.005

[B28] Foster J. M. Hagedorn W. B. (2014). Through the eyes of the wounded: a narrative analysis of children's sexual abuse experiences and recovery process. J. Child Sex. Abus. 23, 538–557. 10.1080/10538712.2014.918072 24819252

[B29] George N. Bance L. O. (2019). Coping strategies as a predictor of post traumatic growth among selected female young adult victims of childhood sexual abuse in Kerala, India. Indian J. Health Well-being 10, 236–240. Retrieved from: http://www.iahrw.com/index.php

[B30] Guggisberg M. (2012). Sexual violence victimisation and subsequent problematic alcohol use: Examining the self-medication hypothesis. Int. J. Arts Sci. 5, 723–736. Available online at: https://www.semanticscholar.org/paper/Sexual-Violence-Victimisation-and-Subsequent-Use%3A-Guggisberg/1b5433ebf78ca19a7b27ca7cb8d5aa01a3e0cf8c

[B31] Guggisberg M. (2017). The wide-ranging impact of child sexual abuse. Curr. Opin. Neurobiol. Sci. 1, 255–264. Available online at: https://www.scientiaricerca.com/srcons/pdf/SRCONS-01-00039.pdf

[B32] Guggisberg M. (2018). The impact of violence against women and girls: a life span analysis, in Violence Against Women in the 21st Century: Challenges and Future Directions, eds Guggisberg M. Henricksen J. (New York, NY: Nova Science Publishers), 3–27.

[B33] Guggisberg M. (2021). Sexuality and sexual health: professional issues for nurses, in Nursing in Australia: Contemporary Professional and Practice Insights, eds Wilson N. J. Lewis P. Hunt L. Whitehead L. (London: Routledge), 201–210. 10.4324/9781003120698-25

[B34] Guggisberg M. Botha T. Barr J. (2021). Child Sexual Abuse Prevention—the Involvement Of Protective Mothers and Fathers: A Systematic Review. J. Fam. Stud. (forthcoming).

[B35] Ha N. Bae S.-M. M.-H. H. (2019). The effect of forgiveness writing therapy on post-traumatic growth in survivors of sexual abuse. Sexual Relationsh. Therapy 34, 10–22. 10.1080/14681994.2017.1327712

[B36] Hafstad G. S. Kilmer R. P. Gil-Rivas V. (2011). Posttraumatic growth among Norwegian children and adolescents exposed to the 2004 tsunami. Psychol. Trauma Theory Res. Pract. Policy 3, 130–138. 10.1037/a0023236

[B37] Hartley S. Johnco C. Hofmeyr M. Berry A. (2016). The nature of posttraumatic growth in adult survivors of child sexual abuse. J. Child Abuse 25, 201–220. 10.1080/10538712.2015.1119773 26934545

[B38] Hawn S. E. Lind M. J. Conley A. Overstreet C. M. Kendler K. S. Dick D. M.. (2018). Effects of social support on the association between precollege sexual assault and college-onset victimization. J. Am. College Health 66, 467–475. 10.1080/07448481.2018.1431911 29405876 PMC6078834

[B39] Herman J. L. (2015). Trauma and Recovery. New York, NY: Basic Books.

[B40] Hitter T. L. Adams E. M. Cahill E. J. (2017). Positive sexual self-schemas of women survivors of childhood sexual abuse. Couns. Psychol. 45, 266–293. 10.1177/0011000017697194

[B41] Hong Q. N. Fabregues Feijoo S. Bartlett G. Cargo M. Dagenais P. Gagnon M.-P.. (2018). The mixed methods appraisal tool (MMAT) version 2018 for information professionals and researchers. Educ. Inform. 34, 285–b291. 10.3233/EFI-180221

[B42] Hong Q. N. Pluye P. Fabregues S. Bartlett G. Boardman F. Cargo M.. (2019). Improving the content validity of the mixed methods appraisal tool: a modified e-Delphi study. J. Clin. Epidemiol. 111, 49–59. 10.1016/j.jclinepi.2019.03.008 30905698

[B43] Kaiser E. Sinanan A. N. (2020). Survival and resilience of female street children experiencing sexual violence in Bangladesh: A qualitative study. J. Child Sex. Abus. 29, 550–569. 10.1080/10538712.2019.1685615 31692413

[B44] Kaye-Tzadok A. Davidson-Ard B. (2016). Posttraumatic growth among women survivors of childhood sexual abuse: its relation to cognitive strategies, posttraumatic symptoms and resilience. Psychol. Trauma 8, 550–558. 10.1037/tra0000103 27018919

[B45] Kelley E. L. Gidycz C. A. (2015). Labeling of sexual assault and the relationship with sexual functioning: the mediating role of coping. J. Interpers. Violence 30, 348–366. 10.1177/0886260514534777 24860074

[B46] Kerlin A. M. Sosin L. S. (2017). Recovery from childhood abuse: a spiritually integrated qualitative exploration of 10 women's journeys. J. Spiritual. Mental Health 19, 189–209. 10.1080/19349637.2016.1247411

[B47] Kirkner A. Ullman S. E. (2019). Sexual assault survivors' posttraumatic growth: Individual and community-led differences. Violence Against Women 26, 1987–2003. 10.1177/1077801219888019 31802694

[B48] Lahav Y. Ginzburg K. Spiegel D. (2019). Post-traumatic growth, dissociation and sexual revictimization in female childhood sexual abuse survivors. Child Maltreat. 25, 96–105. 10.1177/1077559519856102 31248267

[B49] McClain N. Frederick Amar A. (2013). Female survivors of child sexual abuse: finding voices through research participation. Issues Ment. Health Nurs. 34, 482–487. 10.3109/01612840.2013.773110 23875549

[B50] McFerran K. S. Lai H. I. C. Chang W.-H. Acquaro D. Chin T. C. Stokes H.. (2020). Music, rhythm and trauma: a critical interpretive synthesis of research literature. Front. Psychol. 11:324. 10.3389/fpsyg.2020.00324 32180753 PMC7059618

[B51] Mendes K. Keller J. Ringrose J. (2019). Digitized narratives of sexual violence: making sexual violence felt and known through digital disclosures. New Media Soc. 21, 1290–1310. 10.1177/1461444818820069

[B52] Messman-Moore T. L. Long P. J. (2003). The role of childhood sexual abuse sequelae in the sexual revictimization of women: an empirical review and theoretical reformulation. Clin. Psychol. Rev. 23, 537–571. 10.1016/S0272-7358(02)00203-9 12788109

[B53] Moher D. Shamseer L. Clarke M. Ghersi D. Liberati A. Petticrew M.. (2015). Preferred reporting items for systematic review and meta-analysis protocols (PRISMA-P) 2015 statement. BioMed Central 4, 1–9. Available online at: https://systematicreviewsjournal.biomedcentral.com/articles/10.118/2046-4053-4-1. 10.1186/2046-4053-4-1 25554246 PMC4320440

[B54] Moors R. W. R. (2013). The dance of disclosure: online self-disclosure of sexual assault. Qual. Soc. Work 12, 799–815. 10.1177/1473325012464383

[B55] Muldoon O. T. Haslam S. A. Haslam C. Cruwys T. Keams M. Jetten J. (2020). The social psychology of responses to trauma: social identity pathways associated with divergent traumatic responses. Eur. Rev. Soc. Psychol. 30, 311–348. 10.1080/10463283.2020.1711628

[B56] Nelson K. M. Hagedorn W. B. Lambie G. W. (2019). Influence of attachment style on sexual abuse survivors' posttraumatic growth. J. Counsel. Dev. 97, 227–237. 10.1002/jcad.12263

[B57] Nordstrand A. E. Boe H. J. Holen A. Reichelt J. G. Gjerstad C. L. Hjemdal O. (2020). Social support and disclosure of war-zone experiences after deployment to Afghanistan—implications for posttraumatic deprecation or growth. Traumatology 26, 351–360. 10.1037/trm0000254

[B58] Ochoa Arnedo C. Sanchez N. Sumalla E. C. Casellas-Grau A. (2019). Stress and growth in cancer: mechanism and psychotherapeutic interventions to facilitate a constructive balance. Front. Psychol. 10, 1–12. 10.3389/fpsyg.2019.00177 30778323 PMC6369350

[B59] Perez-Gonzalez A. Guilera G. Pereda N. Jarne A. (2017). Protective factors promoting resilience in the relation between child sexual victimization and internalizing and externalizing symptoms. Child Abuse Neglect 72, 393–403. 10.1016/j.chiabu.2017.09.006 28917189

[B60] Pessoa A. S. Colmbra R. M. Bottrell D. Noltemeyer A. (2017). Resilience processes within the school context of adolescents with sexual violence history. Educ. Rev. 33, 1–25. 10.1590/0102-4698157785

[B61] Phanichrat T. Townshend J. M. (2010). Coping strategies used by supervisors of childhood sexual abuse on the journey to recovery. J. Child Abuse 19, 62–78. 10.1080/10538710903485617 20390779

[B62] Phasha T. N. (2010). Educational resilience among African survivors of child sexual abuse in South Africa. J. Black Stud. 40, 1234–1253. 10.1177/0021934708327693

[B63] Saint Arnault D. Sinko L. (2019). Hope and fulfillment after complex trauma: using mixed methods to understand healing. Front. Psychol. 10:2061. 10.3389/fpsyg.2019.02061 31616333 PMC6764148

[B64] Salter M. (2013). Justice and revenge in online counter-publics: emerging responses to sexual violence in the age of social media. Crime Media Cult. 9, 225–242. 10.1177/1741659013493918

[B65] Schick F. (1998). Making Choices: A Recasting of Decision Theory. Cambridge, UK: Cambridge University Press.

[B66] Scoglio A. A. J. Kraus S. W. Saczynski J. Jooma S. Molnar B. E. (2019). Systematic review of risk and protective factors for revictimization after child sexual abuse. Trauma Violence Abuse 22, 41–53 10.1177/1524838018823274 30669947

[B67] Scott K. M. Koenen K. C. King A. Petukhova M. V. Kessler R. C. (2018). Post-traumatic stress disorder associated with sexual assault among women in the WHO World Mental Health Surveys. Psychol. Med. 48, 155–167. 10.1017/S0033291717001593 28625214 PMC5896282

[B68] Serisier T. (2019). A new age of believing women? Judging rape narratives online, in Rape Narratives in Motion, eds Anderson U. Edgren M. Karlsson L. Nilsson G. (Cham, Switzerland: Palgrave Macmillan), 199–222. 10.1007/978-3-030-13852-3_9

[B69] Simon V. A. Smith E. Fava N. Feiring C. (2015). Positive and negative posttraumatic change following childhood sexual abuse are associated with youths' adjustment. Child Maltreatment 20, 278–290. 10.1177/1077559515590872 26092440 PMC5593744

[B70] Singh A. A. Hays D. G. Chung Y. B. Watson L. (2010). South Asian immigrant women who have survived child sexual abuse: resilience and healing. Violence Against Women 16, 444–458. 10.1177/1077801210363976 20224114

[B71] Smigelsky M. A. Gill A. R. Foshager D. Aten J. D. Im H. (2017). My heart is in his hands: the lived spiritual experiences of Congolese refugee women survivors of sexual violence. J. Prev. Interv. Commun. 45, 281–273. 10.1080/10852352.2016.1197754 28880807

[B72] Strauss Swanson C. Szymanski D. M. (2020). From pain to power: an exploration of activism, the #Me Too movement, and healing from sexualt assault trauma. J. Couns. Psychol. 67, 653–668. 10.1037/cou0000429 32212761

[B73] Sweeney A. Filson B. Kennedy A. Collinson L. Gillard S. (2018). A paradigm shift: relationships in trauma-informed mental health services. Br. J. Psychiatry Adv. 24, 319–333. 10.1192/bja.2018.29 30174829 PMC6088388

[B74] Tanaka M. Suzuki Y. E. Aoyama I. Takaoka K. MacMillan H. L. (2017). Child sexual abuse in Japan: a systematic review and future directions. Child Abuse Neglect 66, 31–40. 10.1016/j.chiabu.2017.02.041 28291536

[B75] Tedeschi R. G. Calhoun L. G. (1996). The post-traumatic growth inventory: measuring the positive legacy of trauma. J. Trauma. Stress 9, 455–471. 10.1002/jts.2490090305 8827649

[B76] Tedeschi R. G. Shakespeare-Finch J. Taku K. Caihoun L. G. (2018). Posttraumatic Growth: Theory, Research, and Applications. New York, NY: Routledge. 10.4324/9781315527451

[B77] Ullman S. E. (2014). Correlates of posttraumatic growth in adult sexual assault victims. Traumatology 20, 219–224. 10.1037/h0099402 25379029 PMC4217480

[B78] Ullman S. E. Peter-Hagene L. (2014). Social reactions to sexual assault disclosure, coping, perceived control, and PTSD symptoms in sexual assault victims. J. Community Psychol. 42, 495–509. 10.1002/jcop.21624 24910478 PMC4043331

[B79] Ulloa E. Guzman M. L. Cala C. (2016). Posttraumatic growth and sexual violence: a literature review. J. Aggress. Maltreat. Trauma 25, 286–304. 10.1080/10926771.2015.1079286 29503522 PMC5831550

[B80] Vilencia S. Shakespeare-Finch J. Obst P. (2013). Exploring the process of meaning making in healing and growth after childhood sexual assault: a case study approach. Counseling Psychology Quarterly 26, 39–54. 10.1080/09515070.2012.728074

[B81] Vitek K. N. Yeater E. A. (2020). The association between a history of sexual violence and romantic relationship functioning: a systematic review. Trauma Violence Abuse 2020, 1–12. 10.1177/1524838020915615 32242504

[B82] Volgin R. N. Shakespeare-Finch J. Shochet I. M. (2019). Posttraumatic distress, hope and growth in survivors of commercial sexual exploitation in Nepal. Traumatology 25, 181–188. 10.1037/trm0000174

[B83] Walker H. E. Freud J. S. Ellis R. A. Fraine S. M. Wilson L. C. (2019). The prevalence of sexual revictimization: a meta-analytic review. Trauma Violence Abuse 20, 67–80. 10.1177/1524838017692364 29333937

[B84] Walker-Williams H. J. van Eeden C. van der Merwe K. (2012). The prevalence of coping behavior, posttraumatic growth and psychological wellbeing in women who experienced childhood sexual abuse. J. Psychol. Africa 22, 617–622. 10.1080/14330237.2012.10820576

[B85] Wang Y.-W. Heppner P. P. (2011). A qualitative study of childhood sexual abuse survivors in Taiwan: toward a transactional and ecological model of coping. J. Couns. Psychol. 58, 393–409. 10.1037/a0023522 21574695

[B86] Warner Stidham A. Drauker C. B. Martsolf D. S. Mullen L. P. (2012). Altruism in survivors of sexual violence: the typologty of helping others. J. Am. Psychiatric Assoc. 18, 146–155. 10.1177/1078390312440595 22495915 PMC3947809

[B87] Wasco S. M. (2003). Conceptualizing the harm done by rape: applications of trauma theory to experiences of sexual assault. Trauma Violence Abuse 4, 309–322. 10.1177/1524838003256560 15006299

[B88] Whitelock C. F. Lamb M. E. Rentfrow P. J. (2013). Overcoming trauma: psychological and demographic characteristics of chold sexual abuse survivors in adulthood. Clin. Psychol. Sci. 1, 351–362. 10.1177/2167702613480136

[B89] Williams J. Nelson-Gardell D. (2012). Predicting resilience in sexually abused adolescents. Child Abuse Neglect 36, 53–63. 10.1016/j.chiabu.2011.07.004 22265933

[B90] Wilson D. R. Vidal B. A W. W. Salyer S. L. (2012). Overcoming sequelea of childhood sexual abuse with stress management. J. Psychiatr. Ment. Health Nurs. 19, 587–593. 10.1111/j.1365-2850.2011.01813.x 22070354

[B91] Zinzow H. M. Thompson M. (2015). A longitudinal study of risk factors for repeated sexual coercion and assault in U.S. college men. Arch. Sex. Behav. 44, 213–222. 10.1007/s10508-013-0243-5 24567167 PMC12105606

[B92] Zraly M. Rubin S. E. Mukamana D. (2013). Motherhood and resilience among Rwandan genocide-rape survivors. Ethos 41, 411–439. 10.1111/etho.12031

